# Absence of T-box transcription factor 21 limits neuromuscular junction recovery after nerve injury in *T-bet*-knockout mice

**DOI:** 10.3389/fcell.2025.1535323

**Published:** 2025-03-14

**Authors:** Albina Jablonka-Shariff, Curtis Broberg, Alison K. Snyder-Warwick

**Affiliations:** ^1^ Research Scientist, Division of Plastic Surgery, Department of Surgery, Washington University School of Medicine, St. Louis, MO, United States; ^2^ Research Student, Washington University School of Medicine, St. Louis, MO, United States; ^3^ Division of Plastic Surgery, Department of Surgery, Washington University School of Medicine, St. Louis, MO, United States

**Keywords:** nerve injury, NMJ-neuromuscular junction, terminal schwann cells, immune cells, tbet/Tbx21 transcription factor

## Abstract

**Introduction:**

Terminal Schwann cells (tSCs), at the neuromuscular junction (NMJ), play critical roles in the repair of motor axon terminals at muscle, and rebuild neuronal signaling following nerve injury. Knowledge of mediators impacting tSCs post-nerve injury and in disease may guide beneficial therapies to improve motor outcomes. We previously found T-box transcription factor 21 (TBX21/TBET), classically associated with T-helper1 cells and immune cell recruitment, is expressed in tSCs at the mouse NMJ. The purpose of this study was to examine effects of *Tbx21* absence during NMJ regeneration following peripheral nerve injury.

**Methods:**

Wildtype (WT) and *Tbet-knockout* (*Tbet-KO*) mice underwent sciatic nerve transection and immediate repair. Functional muscle recovery assessment was performed with muscle force testing on mice at 2-, 3-, 4-, and 6-week (wks) and 6 months after nerve injury repair. Morphometric analyses of NMJ reinnervation, tSC number, and tSC processes were evaluated. Full NMJ reinnervation was defined as ≥75% coverage of endplates by axons. A minimum of three mice were evaluated in each group, and 50–100 NMJs were evaluated per mouse.

**Results:**

*Tbet-KO* mice had significantly diminished muscle function compared to WT mice at every time point beyond 3 weeks. *Tbet-KO* mice showed just over half of the muscle force generated by WT mice at 4 weeks and 6 weeks post-injury and repair. By 6 months, *Tbet-KO* mice generated only 84.1% the muscle force of WT mice. *Tbet-KO* mice showed significantly decreased levels of fully reinnervated NMJs compared to WT mice at each time point tested. *Tbet-KO* mice also showed a lower number of tSCs with reduced cytoplasmic processes beyond NMJ area and lower number of immune cells during process of NMJ regeneration.

**Discussion:**

Our findings show that the *Tbx21* transcription factor promotes NMJ reinnervation to regain muscle function following nerve injury.

## 1 Introducton

The peripheral nervous system has the capability to regenerate from trauma or disease, but life-long disability is still common after nerve injury. Functional motor recovery of the end-target muscle after nerve injury depends on two key factors: 1) nerve regeneration at the injury site; and 2) neuromuscular junction (NMJ) reinnervation within target muscle where motor axons correctly match to motor endplates. Axonal regeneration across a nerve transection site has been shown to be largely driven by immune-mediated mechanisms (Cattin et al., 2015; Pan et al., 2019; Zigmond and Echevarria, 2019). The interplay between the NMJ and the immune system is also described after peripheral nerve injuries. We showed previously an increase in macrophage number at the NMJ after nerve transection and immediate repair and observed that inhibition of macrophage recruitment resulted in poor NMJ reinnervation and diminished muscle function (Lu et al., 2020).

The NMJ is the interface between neural input and motor output. The mature NMJ is composed of a single presynaptic unmyelinated terminal nerve, terminal Schwann cells (tSCs), which help to maintain the physical integrity of the synaptic junction, and post-synaptic acetylcholine receptors directly overlying muscle fibers. The NMJ and its components are remarkably stable over the healthy, adult lifespan (O'Malley et al., 1999; Zuo and Bishop, 2008). Upon denervation, phenotypic alterations to NMJ structure occur, and activated tSCs elaborate processes beyond the NMJ following nerve injury (Lu et al., 2020; Kang et al., 2014; Ko and Robitaille, 2015; Vannucci et al., 2019; Jablonka-Shariff et al., 2019). tSCs attract motor axons back to denervated NMJs and provide a path for regenerating axon sprouts from surviving NMJs to reinnervate vacated or new synaptic sites (Reynolds and Woolf, 1992). tSCs also phagocytose the degenerating synaptic components, clearing the way for the reinnervation process (Duregotti et al., 2015; Cunningham et al., 2020). tSC plasticity at NMJs is critical in the restoration of synaptic connections, however the molecular signaling and the transcriptional reprogramming during tSC activation following traumatic peripheral nerve injuries remain elusive.

While many Schwann cell transcription factors contributing to axonal regeneration in the nerve are well described (Patodia and Raivich, 2012; Zhang et al., 2019; Nocera and Jacob, 2020; Zhang et al., 2023), analogous signaling contributing to NMJ reinnervation has not yet been fully recognized. However, tSCs have been found to express transcription factors for molecules involved in NMJ repair (Reynolds and Woolf, 1992; Perez-Gonzalez et al., 2022). Our previous study demonstrated that T-box transcription factor 21 (TBX21 or T-BET) is expressed in tSCs at the NMJ in different muscles in mice (Jablonka-Shariff et al., 2021). In addition, *Tbx21* mRNA expression was highest in the NMJ area compared with NMJ-free muscle regions or with nerve.

Tbx21 was originally described as a transcription factor that controls the T-helper1 (Th1) genetic program in naive CD4^+^ cells. Tbx21 directly activates interferon-gamma (IFN-γ) gene transcription and enhances development of Th1 cells (Szabo et al., 2000; Szabo et al., 2002). In addition, Tbx21 activates many other Th1 cell-specific genes, which range from cytokines, such as TNFα, chemokines like CC-chemokine ligand 3 (Ccl3) and Ccl4, and chemokine receptors.

CXCR3 and CCR5. These are essential for the effective function of many immune cell subtypes (Jenner et al., 2009; Jayaraman, 2013; Yu et al., 2015). In comparison with TBX21 for T_h_1 cells, GATA3 has been found to be the primary transcription factor for Th2 cells. Therefore, during the initial activation of the naïve CD4^+^ T cell, TBX21 and GATA3 gene expression compete to become their respective subtype (Jenner et al., 2009). More recently, Tbx21 transcription factor was shown to be expressed in other cell types, such as in the brain, endometrium, and adipose tissue (Stolarczyk et al., 2014), raising the possibility for a role in coordinating genetic expression and functions outside of the effector cells of the immune system (Faedo et al., 2002; Kawana et al., 2005; Inman et al., 2008). The unique ability of Tbx21 to interface with and coordinate components of the immune system, coupled with its expression at the NMJ, solidifies Tbx21 as an important investigative target following motor nerve injury. The hypothesis of this study was that lack of *Tbx21* would negatively impact NMJ regeneration after the nerve injury. Thus, the goal was to examine the effects of *Tbx21* absence on the NMJ morphology and muscle function following nerve injury using *Tbx21*-knockout (*Tbet-KO*) mouse model.

## 2 Materials and methods

### 2.1 Animals

All experiments were performed on young adult (3-5 months of age) mice of both sexes. Mice were fed chow *ad libitum* and had continuous access to drinking water. Mice were housed in a central animal facility and were maintained pre- and postoperatively in strict accordance with the National Institutes of Health guidelines and according to protocols approved by the institutional animal research ethics committee (IACUC) at Washington University School of Medicine in St Louis, MO, United States.

Wildtype (WT) mice (C57BL/6J; Stock No. 000664), purchased from the Jackson Laboratory (Bar Harbor, ME) and *S100-GFP* transgenic mice, in which the *S100B* promoter drives GFP expression in all glial cells (Magill et al., 2007), were bred in our laboratory. *Tbet-KO* constitutive knockout mice (kindly provided by Dr. Wayne Yokoyama, Washington University, St. Louis, MO) have been previously described (Szabo et al., 2000). Transgenic mice were genotyped by the TAG Center of Transnetyx (Cordova, TN).

### 2.2 Surgical procedure for nerve injury and immediate repair

For functional and morphological assessments, a sciatic nerve injury model was used. WT and *Tbet-KO* mice were anesthetized with intraperitoneal ketamine (50 mg/kg) and dexmedetomidine (0.5 mg/kg) injections. Buprenorphine-SR (1.0 mg/kg) and sterile saline were administered for pain control and hydration, respectively. After anesthesia, mice were kept warm via use of a heating pad. The skin of the right hindlimb was incised 2 mm posterior and parallel to the femur under a standard operating microscope. The sciatic nerve was located and sharply transected 5 mm proximal to the trifurcation. Immediately following transection, the nerve was repaired using microsuture and fibrin glue (Tessel, Baxter, Deerfield, IL) (Vannucci et al., 2019; Jablonka-Shariff et al., 2019). The skin was closed with 6-0 nylon in a simple interrupted fashion. Anesthesia was reversed with Antisedan (0.5 mg/kg), and animals were recovered on a heating pad.

For NMJ-specific RNA expression analyses after nerve injury, the spinal accessory nerve model in *S100-GFP* mice was utilized as the sternomastoid (SM) muscles in the neck have dense, well-defined NMJ clusters. This anatomy allowed separation of centrally located tSC-enriched NMJ band from non-NMJ muscle tissue under a fluorescent dissecting stereomicroscope (Jablonka-Shariff et al., 2021). After anesthesia performed as described above, a cut was made to expose sternomastoid muscle and the spinal accessory nerve, which passes caudally through this muscle. The transected nerve ends were immediately repaired with fibrin glue, and skin was closed with nylon (6–0). For recovery, mice received anesthetic reversal as described above.

### 2.3 Functional muscle recovery assessment

Tetanic muscle force testing of the injured hindlimb was performed to determine the functional impact of *Tbx21* loss on NMJ reinnervation after sciatic nerve transection and acute repair. The extensor digitorum longus (EDL) muscle from WT or *Tbet-KO* mice was used as it has a long tendon, which is required to facilitate *in vivo* attachment of muscle force analysis equipment. Animals were immobilized on an automated functional station (FASt System, red Rock Laboratories, St. Louis, MO). Muscle force testing was performed by electrically stimulating the sciatic nerve and measuring the EDL contraction force utilizing the Red Rocks Laboratory program as described previously (Santosa et al., 2013; Snyder-Warwick et al., 2018). The peak tetanic force was acquired by determining the optimal stimulation amplitude, muscle length, and stimulation frequency for each mouse. Maximum isometric tetanic force was automatically calculated from the resulting sets of recorded force traces. Following assessment, animals were euthanized, the dissected EDL muscle was weighed, and the tetanic specific muscle force (N/cm2) was calculated by dividing the absolute muscle force by the physiological muscle cross-sectional area. A naïve WT mouse was always tested first to establish assay baseline. The tetanic force was assessed at 3-, 4- and 6-week, and 6 months post-sciatic nerve injury. A total of N = 3–7 animals per group were tested.

### 2.4 RNA extraction and quantitative PCR

Total RNA extracted from synapse-rich (NMJ and endplate) and synapse-free sternomastoid muscles were homogenized using a TRIzol and RNeasy MinElute Cleanup Kit (Qiagen, Hilden, Germany). Total extracted RNA was treated with RNase-free DNase (QIAGEN) (Lu et al., 2020). Purity and concentration of RNA were measured using a NanoDrop ND-1000 and only those samples with a 260 nm/280 nm ratio of between 1.6 and 2.0 were used for analysis. Complementary DNA was synthesized from total RNA (100 ng) using High-Capacity cDNA Transverse Transcription kit (Applied Biosystems).

Quantitative real-time PCR (qRT-PCR) was performed using TaqMan Fast Universal PCR Master mix and specific TaqMan PCR primers-probes combination were purchased from Applied Biosystems. Tbx21 (Mm00450960-m1) and Gapdh (Mm99999915-g1) Taq-Man.

Gene Expression Assays were used. All qPCR studies were performed on a Step One Plus instrument (Applied Biosystems), and results were analyzed using Microsoft Excel and quantified using the ΔΔCt method (Livak and Schmittgen, 2001). Relative expression levels were calculated for the *Tbx21* gene by normalizing first to Gapdh level. Samples were run in triplicate, and “non-template controls” with water and no RT enzyme were included as negative controls. Relative gene expression for NMJs on the injured side is shown as fold change from the naïve mouse. The qRT-PCR was analyzed at 2 days, 1 week, 2-, 3-, 4- and 6-weeks post-nerve injury. A total of N = 3-5 animals per group were used.

### 2.5 Muscle harvest, immunofluorescence staining and NMJ analyses

The EDL and tibialis anterior (TA) muscles from right injured hindlimb were dissected and fixed overnight with 4% paraformaldehyde solution (Electron Microscopy Science, Hatfield, PA, United States) and embedded in OCT (Tissue-Tek, Miles, Elkhart, IN, United States). All OCT specimens were stored at −80°C until use. Longitudinal frozen sections (25 μm thick) or whole mount of EDL muscles were processed for immunofluorescence labeling of NMJ components as described previously (Jablonka-Shariff et al., 2019; Snyder-Warwick et al., 2018).

Primary antibodies used for this experiment were as follows: rabbit anti-S100b (Agilent Cat# Z0311, RRID:AB_10013383) for labeling of Schwann cells, a combination of rabbit anti-neurofilament 200 (NF200, Sigma-Aldrich Cat# N4142, RRID:AB_477272) and mouse anti-synaptic vesicle glycoprotein 2 (SV2,DSHB Cat# SV2, RRID:AB_2315387) or mouse anti-2H3 (DSHB Cat# 2H3, RRID:AB_531793) antibodies for labeling axons and axon terminals, mouse anti-Synaptophysin (Abcam Cat# ab8049, RRID:AB_2198854), rat anti-CD68 (Bio-Rad Cat# MCA 1957, RRID:AB_322219), rat anti-CD3 (Thermo Fisher Scientific Cat# 14-0032-85, RRID:AB_467054) and mouse anti-Tbet (Santa Cruz Biotechnology Cat# sc-21749, RRID:AB_628331). After overnight incubation at 4°C, sections were incubated in secondary antibodies conjugated with Alexa Fluor 488 nm, −594 nm or −594 nm (Life Technologies). Tissues were further incubated with α-bungarotoxin (α-BTX) Alexa Fluor 555 (Thermo Fisher Scientific Cat# B35451, RRID:AB 2617152) or −647. α-BTX binds specifically to AChRs in the postsynaptic membrane. Control sections (either no primary or no secondary antibody) were included in every type of staining to test for nonspecific staining and autofluorescence. Slides were prepared using Vectashield mounting medium with DAPI (Vector Laboratories Cat# H-1200, RRID:AB_2336790) to label nuclei.

For whole mounting of the EDL muscles all four bellies were imaged, analyzed and averaged to obtain a value for each single animal (n = 1). A minimum of 20-30 NMJs were acquired per animal. For muscle sections at least five random sections and 50-100 NMJs per muscle/animal were analyzed. A total of N = 3-7 animals per group were used. Morphological evaluation of NMJ components were performed in a blinded fashion by two investigators unless otherwise noted as described previously (Jablonka-Shariff et al., 2019; Snyder-Warwick et al., 2018). To determine the number of tSCs/NMJ, the percentage of tSCs showing processes and the percentage of NMJs with tSCs, muscle sections were stained with S100 antibody and α-BTX. To evaluate NMJ innervation, sections or whole mounts (EDL) were stained using a combination of NF200/SV2 antibodies, which label neurofilaments/synaptic vesicles, or NF200/2H3 antibodies and α-BTX for endplates. An NMJ was defined as fully innervated if the nerve terminal and endplate aligned ≥75% of their length. The percentage of innervated NMJs was normalized to the total number of NMJs in the macroscopic fields of view. Denervation was defined as no overlap of neurofilaments and α-BTX staining.

The number of CD68^+^ macrophages and CD3^+^ T cells stained with rat anti-CD68 and rabbit anti-CD3 antibodies were counted per field of microscopic view. The imaging was focused on fields containing NMJs along with visualization of the surrounding areas.

For quantitative analyses, all NMJs were imaged using the Fluoview FV1000 confocal microscope (Olympus, Waltham, MA) and an Axio Imager M2 fluorescent microscope (Zeiss). *En-face* images of NMJs were analyzed using NIH ImageJ macro (https://imagej.nih.gov/ij/). The fluorescence intensity for synaptophysin/NMJ in naïve WT and *Tbet-KO* mice was calculated as follows: Integrated Density – (area of NMJ x mean fluorescence of background values). Figures were prepared using Adobe Photoshop CC 2025 and Adobe Illustrator CC 2025 system (Adobe Systems, San Jose, California).

### 2.6 Statistical analysis

Continuous variables are reported as mean ± SD and categorical variables in percentages. Significance is set as *P* < 0.05. Quantification of reinnervation, tSCs, and immune cells were performed in a blinded fashion with two evaluators. Statistical analyses were performed by two-tailed, unpaired Student’s *t* tests or Mann-Whitney U tests using Microsoft Excel and GraphPad Prism 10 (GraphPad Software).

## 3 Results

### 3.1 *Tbx21* mRNA expression increases at NMJs in WT mice after nerve injury

**FIGURE 1:**
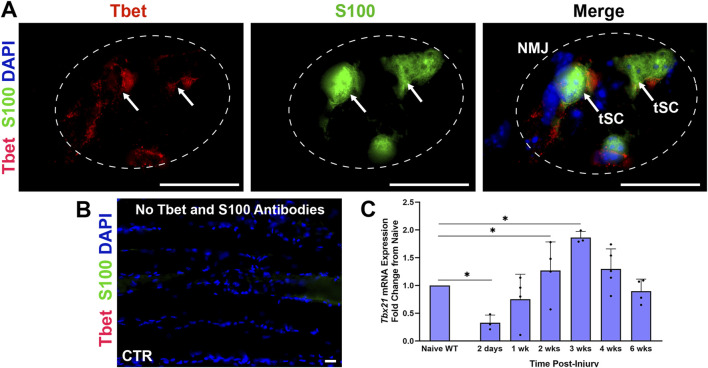
*Tbx21* mRNA expression increases at the neuromuscular junction (NMJ) in young adult wildtype *(*WT) mice following peripheral nerve transection and immediate repair **(A)**. Representative image showing colocalization of Tbet immunostaining (red) with terminal Schwann cells (tSCs, green, arrows) at the NMJ in the Extensor Digitorum Longus (EDL) muscle from young adult naïve *S100-GFP* mice **(B)**. No Tbet or S100 is observed at the NMJs in the muscle without primary antibodies (control, CTR) **(C)**. *Tbx21* mRNA expression at the NMJ is highest at 3 weeks (wks) post-injury compared to NMJ from naïve uninjured wildtype (WT) mice of both sexes. Tbet Ab = Tbet positive tSC at the NMJ; S100 Ab = glial cells (green), DAPI = nuclear staining (blue). Scale bar = 20 μm **(A–B)**. Data: Mean ± SD **(C)**; N = 3-5 mice; *P < 0.05.

We reported previously that Tbet (Tbx21) protein is localized in tSCs at NMJs within skeletal muscles in homeostasis (Figures 1A, B) and demonstrated that *Tbx21* mRNA expression was over nine-fold higher in the NMJ area relative to synapse-free sternomastoid muscle or sciatic nerve in the mouse (Jablonka-Shariff et al., 2021). Given the robust immune response within the muscle of the WT mice following nerve injury, we first assessed the expression of *Tbx21* mRNA at the NMJ following sciatic nerve injury and immediate repair in *S100-GFP* mice (Figure 1C). *Tbx21* gene expression, shown via qPCR, increases from 2 weeks to 4 weeks after nerve injury compared to naïve uninjured mice. The mRNA levels significantly peaked at 2 and 3 weeks (*P* < 0.0001 and *P <* 0.05, respectively) post-injury, suggesting a potential role of *Tbx21* in NMJ regeneration.

### 3.2 Young adult *Tbet-KO* mice display normal NMJ morphology in homeostasis

**FIGURE 2:**
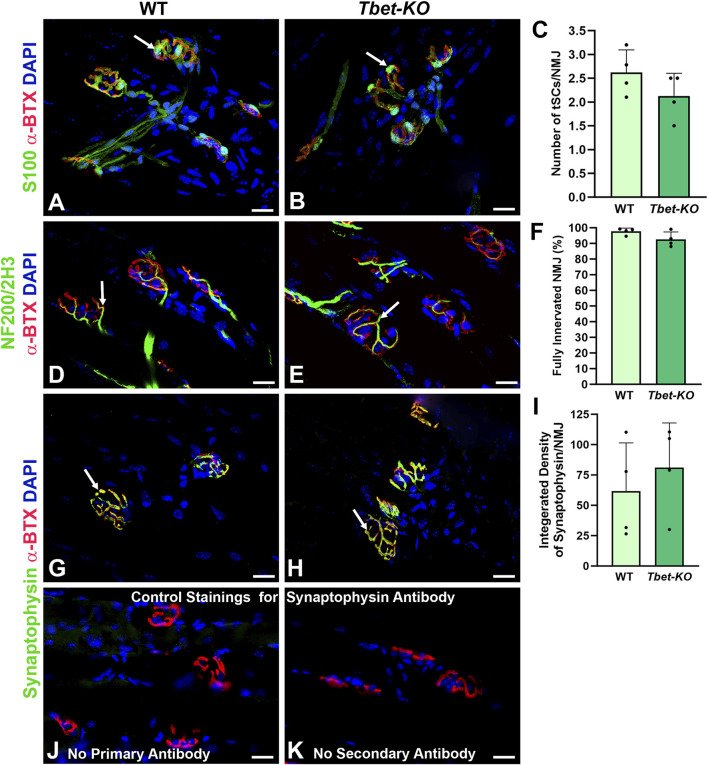
The neuromuscular junctions (NMJs) of naïve *Tbet-KO* young adult mice of both sexes show no morphological abnormalities **(A–C)**. NMJs from WT and *Tbet-KO* mice showing 2-3 tSCs stained with anti-S100 antibody (green, arrows) localized over endplates (red) in the EDL muscle **(D–F)**. *Tbet-KO* naïve mice show similar proportion of fully innervated NMJs stained with NF200/2H3 antibodies (Abs, green, arrows) compared to naïve WT mice. NMJs were defined as fully innervated if nerve occupied ≥75% of endplate (red) **(G–I)**. Synaptic vesicle intensity at the NMJs stained with anti-synaptophysin Ab (green, arrows) is comparable in naive WT and *Tbet-KO* mice **(J–K)**. Control staining for synaptophysin monoclonal Ab; no staining is observed with no primary **(J)** or secondary **(K)** Abs is observed at the NMJs. NF200/2H3 Abs = axons/nerve terminal (green); α-BTX = α-bungarotoxin for Acetylcholine receptors (AChRs, red); DAPI = nuclear staining (blue). Data: Mean ± SD **(C, F, I)**; N = 4 mice; Scale bar = 20 μm **(A–B, D-E, G-H, J-K)**.

EDL and TA muscle synaptic morphology from young adult (3–5-month-old) *Tbet-KO* mice showed similar NMJ structure to naïve control WT mice. There were no differences in mean tSC number per NMJ (2.1 ± 0.4 tSCs per NMJ in *Tbet-KO* mice vs. 2.6 ± 0.5 in WT mice), which stayed in contact with the entire endplate and expressed S100 antibody, a marker of the Schwann cells (Figures 2A–C). NMJs from *Tbet-KO* mice were fully innervated with nerve terminals (NF200/2H3) overlapped with pretzel-shaped endplates (α-BTX for AChRs), and their morphologies did not differ from WT controls (Figures 2D–F). NMJs also showed similar staining intensity with synaptophysin in synaptic vesicles (Figures 2G–I). No synaptophysin staining is observed at the NMJs with no primary or secondary antibodies (Figures 2J,K).

### 3.3 NMJ reinnervation and muscle function are impaired in *Tbet-KO* mice after nerve transection and immediate repair

**FIGURE 3:**
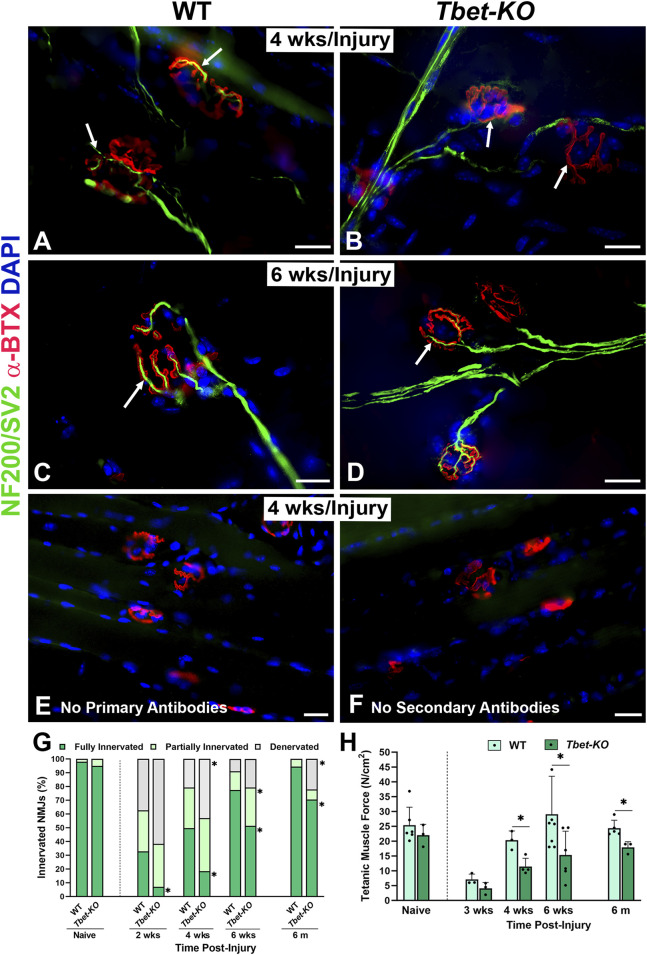
Lack of Tbx21 transcription factor impairs the reinnervation of NMJ and diminished muscle function following sciatic nerve transection and immediate repair **(A–D)**. Representative images of NMJ innervation (arrows) in the EDL muscle from WT and *Tbet-KO* mice 4 weeks (wks, **A, B**) and 6 weeks **(C–D)** following nerve injury. No staining is observed at the NMJs without primary NF200/SV2 **(E)** or secondary antibodies **(F, G)**. Bar graph summarizes the quantitative NMJ findings according to full innervation, partial innervation, or denervated (no innervation) NMJs. NMJs were defined as fully innervated if nerve occupied ≥75% of endplate area. NMJ percentages were normalized to the total NMJ number in the microscopic field of view **(H)**. Bar graph summarizes EDL tetanic muscle force elicited by sciatic nerve stimulation, which was significantly lower in *Tbet-KO* mice compared to injured WT mice. NF200/SV2 Abs = axons/synaptic vesicles (green), α‐BTX = α‐bungarotoxin for AChRs (endplates, red), DAPI = nuclear staining (blue). Scale bars = 20 μm **(A–F)**. Data: Mean ± SD **(G–H)**; N = 3-7 mice per group; *P < 0.05.

NMJ reinnervation in the EDL and/or TA muscles was examined in *Tbet-KO* and control WT mice. Fluorescently stained longitudinal frozen muscle sections and EDL whole mounts were evaluated for NMJ innervation at 2-, 4-, and 6-week and 6 months following sciatic nerve transection and immediate repair. Morphological differences were noted in reinnervation patterns between *Tbet-KO* and control groups (Figure 3). Most notably, neural branching patterns at the NMJs of *Tbet-KO* mice were more disorganized at earlier time points in both groups. This morphology persisted until 4 weeks in the *Tbet-KO* mice (Figure 3). By 6 weeks, the majority of NMJs in the WT mice showed an organized pattern of nerve terminal overlying the endplates while the disorganized pattern often persisted in the *Tbet-KO* group (Figures 3A–D). No staining is observed at the NMJs with no primary or secondary antibodies (Figures 3E, F). Quantitatively, at every time point, *Tbet-KO* mice showed significantly lower percentages of fully innervated NMJs compared to WT mice (Figure 3G). Finally, *Tbet-KO* mice showed decreased (*P <* 0.05) percentages of fully innervated NMJs (70.5% ± 24.6%) and more denervated NMJs compared to WT mice (94.4% ± 13.6%) at 6 months following nerve injury.

Tetanic muscle force analyses were conducted on *Tbet-KO* and WT mice at 3-, 4-, and 6-week, and 6 months following sciatic nerve injury and immediate repair (Figure 3H). At 3 weeks, the tetanic muscle force generated by the *Tbet-KO* mice was 37.1% of WT mice. While the muscle force increased at 4 weeks and at 6 weeks, it remained significantly lower in *Tbet-KO* mice at 6 months compared to WT mice. These data suggest a role for *Tbx21* in the reinnervation process following nerve injury.

### 3.4 Terminal Schwann cells are fewer in number in *Tbet-KO* mice after nerve injury

**FIGURE 4:**
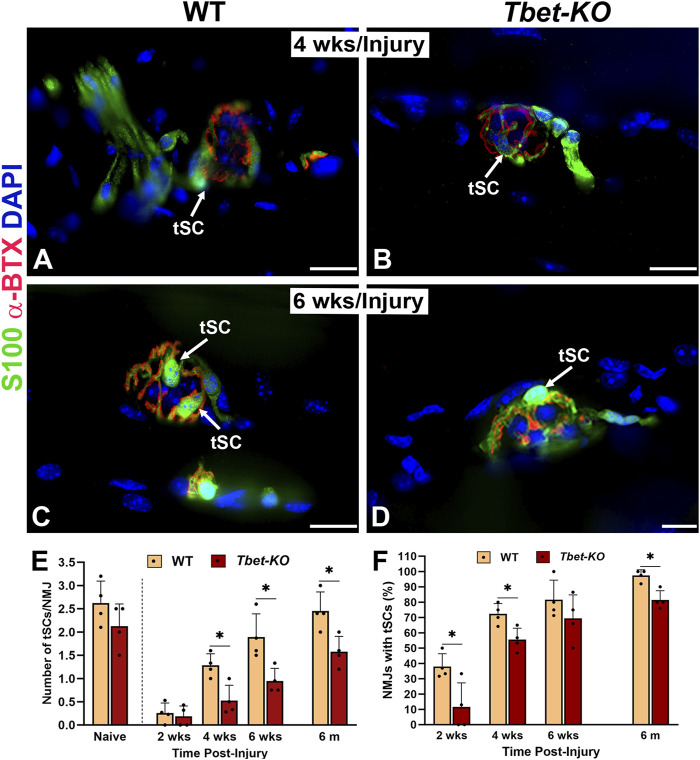
Lack of Tbx21 transcription factor impairs the regeneration of terminal Schwann cells (tSCs) at the NMJ of young adult *Tbet-KO* mice following sciatic nerve transection and immediate repair **(A–D)**. Representative images of tSCs (green, arrows) at the NMJ in the EDL muscle from WT and *Tbet-KO* mice 4 weeks (wks, **A, B**) and 6 weeks **(C–D)** post-nerve injury **(E)**. Bar graph summarizes the number of tSC bodies/NMJ at different time points following injury **(F)**. Bar graph showing the percentage of NMJ with tSCs. S100 Ab = glial cells (green), α‐BTX = α‐bungarotoxin for AChRs (endplates, red), DAPI = nuclear staining (blue). Scale bars = 20 μm **(A–D)**. Data: Mean ± SD **(E–F)**; N = 4 mice per group; *P < 0.05.

tSCs encapsulate the NMJ and play supporting roles in NMJ reinnervation and muscle regeneration after nerve injury. Following nerve injury, NMJs in the hindlimb injured muscles from *Tbet-KO* mice exhibited significantly decreased numbers of tSCs per NMJ (*P* < 0.05) compared to those of WT mice. tSC numbers were reduced to half those of WT at 4 weeks and 6 weeks (Figures 4A–E). Finally at 6 months only 1.6 ± 0.6 of tSCs/NMJ were noted in *Tbet-KO* mice compared to 2.4 ± 0.4 of tSCs/NMJ for WT mice. In addition, *Tbet-KO* mice showed significantly lower percentages of NMJs with tSCs present compared to WT mice (Figure 4F).

### 3.5 Terminal Schwann cell response to nerve injury is altered in *Tbet-KO* mice

**FIGURE 5:**
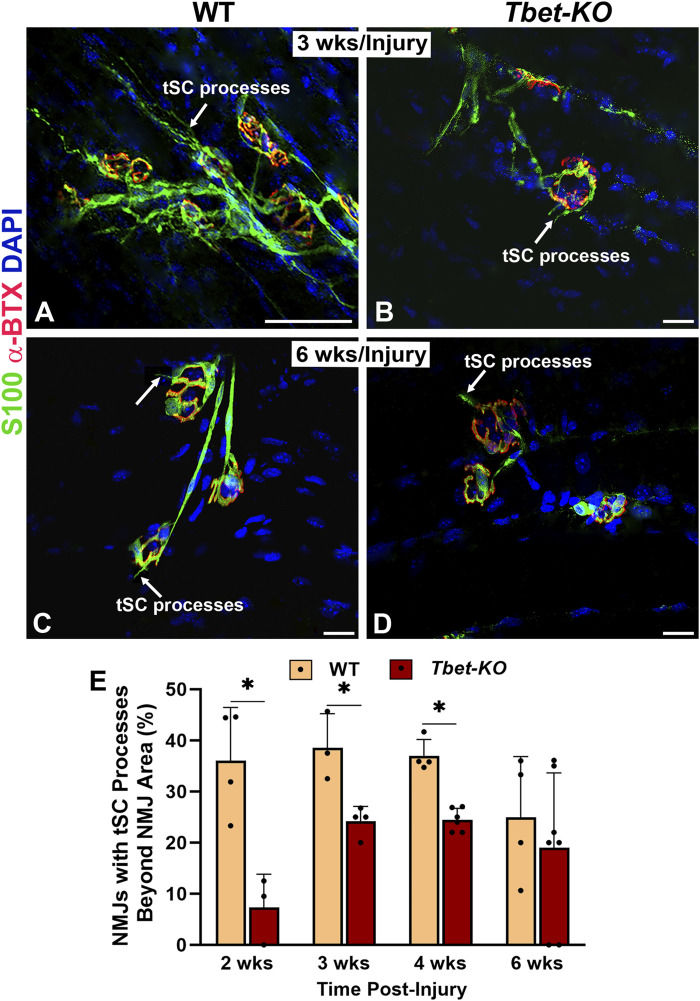
*Tbet-KO* mice show reduced terminal Schwann cell (tSC) process extension beyond the NMJ area in young adult *Tbet-KO* mice after sciatic nerve transection and immediate repair **(A–D)**. Representative images of tSCs (green, arrows) with cytoplasmic processes at the NMJ in the EDL muscle from WT and *Tbet-KO* mice 3 weeks (wks, A-B) and 6 weeks **(C–D)** following nerve injury **(E)**. Bar graph showing the percentage of NMJ with tSC processes, which are reduced and shorter in *Tbet-KO* mice compared to WT mice. S100 Ab = glial cells (green), α‐BTX = α‐bungarotoxin for AChRs (endplates, red), DAPI = nuclear staining (blue). Scale bars = 20 μm **(A–D)**. Data: Mean ± SD **(E)**; N = 3-7 mice per group; *P < 0.05.

Following nerve injury, tSCs normally extend, in advance of axon sprouts, cytoplasmic processes beyond the NMJ in search of neighboring, innervated NMJs. In *Tbet-KO* mice, the percentage of tSCs extending processes was significantly less than that of injured control WT mice during 4 weeks of NMJ regeneration after nerve transection and repair (Figure 5). In addition, tSC processes were short with less extension beyond the NMJ (Figures 5B, D, E). Of note, no tSC processes were observed in the absence of injury (naïve WT and *Tbet-KO* in Figures 2A, B). These results suggest *Tbx21* transcription factor in tSCs plays an important role in tSC morphology and their response to nerve injury, likely affecting synapse remodeling post-injury.

### 3.6 The inflammatory response is altered at the NMJ in *Tbet-KO* mice after nerve injury

**FIGURE 6:**
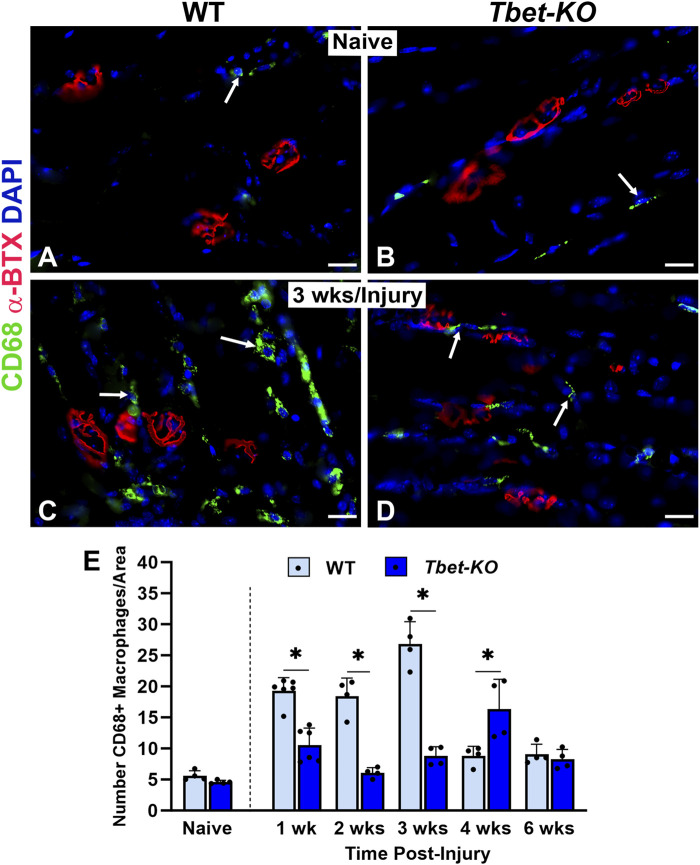
Macrophage numbers are reduced at the NMJ in young adult *Tbet-KO* mice, but not in WT mice, after sciatic nerve transection and immediate repair **(A–D)**. Representative images of CD68^+^ macrophages (green, arrows) around NMJs stained with a‐BTX (red) for endplates in the EDL muscle from naïve, uninjured WT and *Tbet-KO* mice **(A–B)** and at 3 weeks (wks, **C, D**) following nerve injury **(C–D, E)**. Bar graph showing significantly lower macrophage numbers in *Tbet-KO* mice during the first 4 weeks of regeneration compared to WT mice where macrophages peak at 3 weeks post-injury. CD68 Ab = macrophages (green), α‐BTX = α‐bungarotoxin for AChRs (endplates, red), DAPI = nuclear staining (blue). Scale bars = 20 μm **(A–D)**. Data: Mean ± SD **(E)**; N = 4-6 mice per group; *P < 0.05.

**FIGURE 7:**
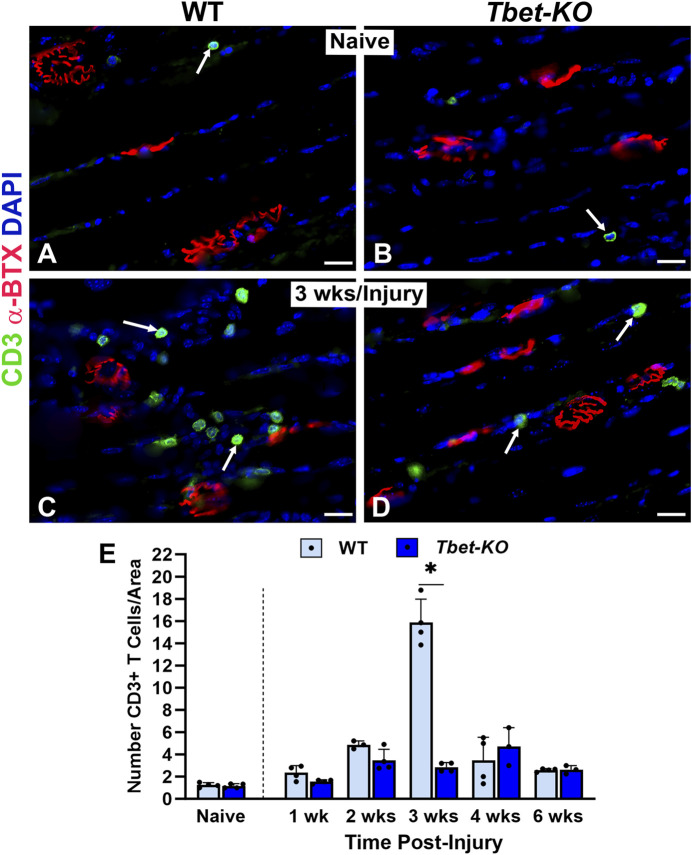
T cell numbers are reduced at the NMJ in young adult *Tbet-KO* mice, but not in WT mice, after sciatic nerve transection and immediate repair **(A–D)**. Representative images of CD3^+^ T cells (green, arrows) around NMJs stained with α‐BTX (red) for endplates in the EDL muscle from naïve, uninjured WT and *Tbet-KO* mice **(A, B)** and at 3 weeks (wks, **C, D**) following nerve injury **(E)**. The number of T cells in WT mice peaks in number at 3 weeks following nerve injury, in contrast to *Tbet-KO* mice which exhibit low T cell numbers at all time points post-injury. CD3 Ab = T cells (green), α‐BTX = α‐bungarotoxin for AChRs (endplates, red), DAPI = nuclear staining (blue). Scale bars = 20 μm **(A–D)**. Data: Mean ± SD **(E)**; N = 3-5 mice per group; *P < 0.05.

The immune response has been shown to be integral in sciatic nerve regeneration and at NMJs in muscle after nerve injury. During NMJ regeneration, macrophages play many critical functions, including orchestration of the inflammatory response and clearance of nerve terminal debris. We found that CD68^+^ macrophages were significantly elevated in WT mice during the first 4 weeks after nerve injury and immediate repair (Figure 6), reaching peak (26.8 ± 3.6) numbers at 3 weeks post-injury (*P* < 0.001). In contrast, *Tbet-KO* mice showed fewer macrophage numbers with a brief and delayed increase in number (16.4 ± 4.8) at 4 weeks post-injury (*P* < 0.05). Similar to macrophages, the number of CD3+T cells was low at all experimental time points in *Tbet-KO* mice compared to WT mice, which showed significantly (*P* < 0.01) elevated T cell numbers (15.9 ± 2.1 WT vs. 2.9 ± 0.4 *Tbet-KO*) at 3 weeks post-injury (Figure 7).

## 4 Discussion

In the nervous system, transcription factors are associated with the cellular fate of injured neurons, the regeneration of axons, and the repair of injured peripheral nerves (Patodia and Raivich, 2012; Zhang et al., 2019; Nocera and Jacob, 2020). They bind to DNA sequences, modulate the transcription of target genes, and regulate various biological processes. Previous work has uncovered several epigenetic and transcriptional effectors implicated in regulating synaptic protein dynamics in health and disease (Li et al., 2012), and during NMJ remodeling after nerve injury (Guzman et al., 2023). Here we revealed a novel function of Tbx21 transcription factor beyond its recognized role in T cell function. Our work shows that Tbx21 plays a positive role at the NMJ after nerve injury. Lack of Tbx21 in *Tbet-KO* mice delayed NMJ reinnervation, NMJ regeneration, and efficient function of end-target muscle after peripheral nerve transection and immediate repair.

Tbx21, originally described as the master regulator of Th1 cell development, drives the expression of molecules such as interferon-gamma (IFN-γ) in effector cells (Szabo et al., 2000; Jenkins et al., 2023), which promotes muscle healing, in part, by stimulating formation of new muscle fibers, and is required for efficient skeletal muscle regeneration (Cheng et al., 2008). We have shown previously that Tbx21 protein is colocalized with non-myelinating tSCs and their processes at the NMJ in young adult WT and *S100-GFP* naïve mice (Jablonka-Shariff et al., 2021). Although the function of Tbet as a cytoplasmic protein remains unknown, differential Tbet localization has been linked to effector Th1 cell status (McLane et al., 2013), activation (Neurath et al., 2002), cell cycle (Chang et al., 2011), and protein stability (Jang et al., 2013). In addition, *Tbx21* mRNA expression was also significantly higher at the NMJ area compared with NMJ-free muscle or nerves in naïve *S100-GFP* mice (Jablonka-Shariff et al., 2021). In the current study, we show an increased *Tbx21* mRNA expression in *S100-GFP* mice after peripheral nerve injury compared to naïve mice. The *Tbx21* gene has been also identified previously after sciatic nerve crush injury (Zhang et al., 2019). Despite the recognition of transcription factors in the nerve and the muscle after nerve injury, our knowledge of the involvement Tbx21 in the muscle after nerve injury is still limited.

To obtain a better understanding of the involvement of Tbx21 transcription factor, we used *Tbet-KO* mice to dissect its role in NMJ reinnervation and muscle regeneration after sciatic nerve transection and immediate repair. We observed a significantly lower number of tSCs per NMJ and decreased percentages of NMJs with tSCs in *Tbet-KO* mice compared to WT mice following nerve repair. It has been shown by others and us that after nerve injury in WT mice, activated tSCs perceive the damage occurring to the motor axon and respond by displaying extensive cytoskeletal reorganization with long processes along which regenerating nerve terminals extend sprouts (Lu et al., 2020; Reynolds and Woolf, 1992; Son et al., 1996; Kang et al., 2003). Without these tSC processes, NMJ reinnervation and muscle function are negatively impacted. To this end, knowledge about the signaling pathways mediating tSC activation and process extension after injury is not described well. However, it has been previously reported that alarmins released by mitochondria of degenerating axon terminals activate tSCs (Duregotti et al., 2015; Cunningham et al., 2020). In addition, the transcription factor c-Jun may also contribute to their activation and promote the sprouting and guidance of regenerating axons back to denervated NMJs (Belotti and Schaeffer, 2020). Considering the localization of Tbx21 in tSCs, this transcription factor may also play a potential role in tSC activation to change their cellular morphology by extending cytoplasmic processes, and this possibility will be further investigated. It is well established that certain sets of transcription factors or proteins can instruct cells to undergo complex cytoskeletal changes (Xu et al., 2015; Negro et al., 2018).

During nerve terminal degeneration, tSCs become phagocytic and together with macrophages (Zigmond and Echevarria, 2019; Duregotti et al., 2015), but not neutrophils (Li et al., 2012; Balog et al., 2023), remove nerve debris, thus permitting functional nerve regeneration. These data suggest that Tbx21 can also coordinate neuroinflammation and upregulation of several cytokines and chemokines required for successful reinnervation at the NMJ after peripheral nerve injury. However, the exact mechanisms and effects of Tbx21 on tSC activation and chemokine upregulation during NMJ reinnervation require further investigation. The present results suggest that Tbx21 may act as a signaling molecule at the skeletal NMJ, mediating presynaptic structural plasticity after nerve injury and potentially representing a therapeutic intervention.

This study also demonstrated a significant decrease in the neuron’s ability to reinnervate NMJs following injury in *Tbet-KO* mice. Namely, we showed significantly lower percentages of fully reinnervated NMJs in *Tbet-KO* mice compared to WT mice at each time point tested. Furthermore, the tetanic muscle force assessment recapitulates the innervation findings, indicating that muscle function is indeed reduced in the absence of *Tbx21* following nerve injury. These results suggest that Tbx21 localized in tSCs may also promote NMJ regeneration. As Schwann cells can be considered as immune-competent cells that recognize antigen and regulate the immune response (Meyer zu Hörste et al., 2008), the local interactions suggest that non-myelinating Schwann cells may have effects on the innate/adaptive immune response (*e.g*., activation of T cells with presented antigen) and *vice versa*. Thus, depending on the context and the environment, Tbx21 may have not only detrimental but also beneficial effects on neuronal survival and function (Schetters et al., 2017). Th1-producing Tbx21 needs to be balanced by other types of helper T cells, such as Th2, Th17 and regulatory T (Treg) cells, to prevent excessive inflammation and tissue damage (Schetters et al., 2017; Astarita et al., 2023). More research is needed to understand how Tbx21 influences the balance between neurodegeneration and neuroprotection in different diseases and conditions.

Given that the immune response plays an important role in NMJ regeneration we evaluated macrophages and CD3^+^ T cells in *Tbet-KO* mice after nerve injury. We show that the numbers of both analyzed immune cells at the NMJ following nerve injury are altered in the muscle of *Tbet-KO* mice. There are fewer macrophages and CD3^+^ T cells around NMJ compared to WT mice after nerve injury. During NMJ and muscle regeneration, macrophages have many critical functions, including orchestration of the inflammatory response, clearance of cellular debris, and regulation of SC proliferation (Zigmond and Echevarria, 2019; Lu et al., 2020). While the leukocytes that initiate and orchestrate inflammatory responses are tissue-specific and are still an area of active study, macrophages and T cells have been shown to be critical to achieving functional regeneration during nerve and muscle regeneration. Tbx21 directs T-cell homing to pro-inflammatory sites by the regulation of CXCR3 expression, which in turn is responsible for T cell differentiation and behavior (Harms et al., 2015; Pritchard et al., 2019). It has been also suggested that Tbx21 has diverse and complex roles in regulating the interactions between T cells and other cells in the nervous system and the immune systems. Tbx21 may play a role in nerve regeneration by regulating the type and function of T cells involved in this process. Schwann cells can also modulate the immune response in the peripheral nervous system by secreting cytokines and chemokines that attract or repel T cells. On other hand, T cells can also affect glial cell activation, differentiation, and survival. For example, Tbx21-expressing Th1 cells can induce microglial activation and neuroinflammation in neurodegenerative diseases such as Alzheimer’s disease and multiple sclerosis. However, Tbx21-deficient Th1 cells can promote oligodendrocyte differentiation and remyelination in a mouse model of demyelination (Racke et al., 2014; Benakis et al., 2022). Tbx21 is also recognized to have a role in other tissues such as brain and endometrium (Faedo et al., 2002; Inman et al., 2008). Th1 immunity, characterized by immune-inflammatory responses, becomes dominant during the peri-implantation period and shows controlled benefits rather than harm. Quickly after the placental implantation, the early inflammatory Th1 immunity is shifted to the Th2 anti-inflammatory immune responses (Wang et al., 2019). It has been also suggested that Tbx21 plays a role in metabolic regulation in adipose tissue however, the downstream molecular interactions through which Tbx21 is able to impact on adiposity and insulin sensitivity are unknown (Stolarczyk et al., 2014).

To our knowledge, this is the first description of Tbx21 as an immune molecule in glial cells influencing nerve regeneration. Our findings reveal a surprising role for the Tbx21 transcription factor in NMJ remodeling after injury. Being a key modulator of gene expression, Tbx21 may play critical roles in converting injury-induced signals to protein alterations and phenotypic changes. The action of Tbx21 may also be greatly influenced by its collaborative molecular partners such as Runx3 and INF-γ (Djuretic et al., 2007). Further work must be done using conditional *T-bet KO* model in tSCs to understand the mechanisms by which *Tbx21* induces these effects, and its specific expression in tSCs indicates that these cells’ other molecule(s) may be critical to this process.

## Data Availability

The raw data supporting the conclusions of this article will be made available by the authors, without undue reservation.
